# *N*-Acetylcysteine Inhibits Platelet Function through the Regeneration of the Non-Oxidative Form of Albumin

**DOI:** 10.3390/antiox11030445

**Published:** 2022-02-23

**Authors:** Sonia Eligini, Benedetta Porro, Giancarlo Aldini, Susanna Colli, Cristina Banfi

**Affiliations:** 1 Centro Cardiologico Monzino I.R.C.C.S., 20138 Milan, Italy; sonia.eligini@cardiologicomonzino.it (S.E.); benedetta.porro@cardiologicomonzino.it (B.P.); 2 Dipartimento di Scienze Farmaceutiche, Università degli Studi di Milano, 20133 Milan, Italy; giancarlo.aldini@unimi.it; 3 Dipartimento di Scienze Farmacologiche e Biomolecolari, Università degli Studi di Milano, 20133 Milan, Italy; susanna.colli@unimi.it

**Keywords:** *N*-acetylcysteine, albumin, platelets, aggregation, arachidonic acid metabolites, adhesion, reactive oxygen species, calcium mobilization

## Abstract

*N*-acetylcysteine (NAC) is able to break down protein disulfides, generating free thiols. This mechanism occurs on mixed disulfides of albumin (HSA) to form mercaptoalbumin (HMA), the main antioxidant species in the plasma. Circulating HSA exists in two main forms: the reduced form (HMA), and the oxidized forms, whose predominant modification is cystenylation (HSA-Cys). Increased levels of oxidized HSA have been detected in several diseases associated with oxidative stress. This study showed that NAC inhibits platelet aggregation by restoring HMA. In addition, the regeneration of HMA by NAC inhibits platelet functions such as intracellular calcium mobilization, reactive oxygen species generation, arachidonic acid metabolites synthesis, and adhesion to the collagen matrix. In our conditions, the exposure of platelets to NAC did not increase GSH levels. However, the inhibition of platelet aggregation was also detected following treatment of platelet-rich plasma with GSH, which, similarly to NAC, reduced HSA-Cys levels. Furthermore, this study showed that cysteine, another compound able to restore HMA by reducing the HSA-Cys content, inhibited platelet aggregation to a similar extent as NAC. The results obtained in this study suggest a new mechanism by which NAC can modulate platelet activation and suggest its possible use as an antiplatelet drug in conditions associated with oxidative stress.

## 1. Introduction

*N*-acetylcysteine (NAC) is a widely used drug in clinical practice as a mucolytic [1] and detoxifying agent against acetaminophen poisoning [2]. Its clinical use is expanding [3,4,5,6,7,8]; for example, it is also used for renal protection in contrast-induced nephropathy [9], as a preventive agent for atrial fibrillation [10], and in the treatment of psychiatric and neurological disorders [11].

In a recent review, the principal molecular mechanisms regarding the antioxidant and reducing activity of NAC have been critically revised [12]. Several in vitro and in vivo studies have shown that NAC induces the intracellular biosynthesis of glutathione (GSH), a well-known direct antioxidant and substrate for several antioxidant enzymes [13]. However, despite the efficacy of NAC being mainly attributed to its antioxidant-dependent ability to generate GSH, all the mechanisms underlying its activity are not yet fully understood in that they may not depend exclusively on GSH replenishment [14,15]. It should be noted, indeed, that the bioavailability of NAC is very low because at physiological pH, the carboxyl group loses its proton, becoming negatively charged, thus reducing the ability of NAC to cross the cell membrane [16]. In some conditions characterized by depletion of endogenous cysteine and GSH, NAC can also act as a direct antioxidant agent for some oxidant species such as nitrogen dioxide and hypohalous acid [12]. According to an additional mechanism recently proposed, NAC is able to break thiol proteins, thus leading to the release of free thiols, which possess superior antioxidant activity than NAC. An example is given by the mixed disulfides of albumin (HSA) whose reaction with NAC generates mercaptoalbumin (HMA), which has an important direct antioxidant activity [12]. It is known, indeed, that HSA exists in two main forms, depending on the redox status of Cys34: the reduced form or HMA, and the oxidized forms that can be further classified as reversible (mixed disulfides of Cys34 with low molecular weight thiols) and irreversible (sulfinic or sulfonic acid derivatives of Cys34) forms [17]. The ratio between the reduced and oxidized forms has important physio-pathological consequences [18,19]. Approximately 70–80% of total HSA in the plasma of healthy subjects is present as the reduced form while about 20–30% forms a disulfide with several thiol compounds such as cysteine, homocysteine, or GSH, and a minor fraction is in an irreversible form (sulfinic and sulfonic states) [18]. Among the mixed disulfide forms of HSA, the predominant modification is cystenylation (HSA-Cys) [18]. A marked increase in the oxidized forms has been reported in several pathological conditions associated with oxidative stress [18]. Indeed, HSA is not only the predominant plasma protein, but is involved in several physiological functions due to its antioxidant, anti-inflammatory, anti-coagulant, and anti-platelet properties [20]. Although it has long been known that HSA inhibits platelet aggregation [21,22,23], it was only recently shown that oxidative modifications convert albumin into a platelet activator [24,25].

Platelets, the smallest circulating cells, play a crucial role in hemostasis. However, they are also actively involved in several cardiovascular disease-related conditions such as atherosclerosis, myocardial infarction, and stroke. Indeed, following endothelial damage, platelets adhere to exposed subendothelial proteins, triggering platelet activation consisting of a shape change, the release of granule contents, and aggregation, which ultimately participates in thrombus formation [26].

In this regard, it has been shown that NAC impairs platelet functions [27,28], but the mechanism(s) through which this phenomenon occurs is not clear [29], and several hypotheses have been formulated [27,28,30]. 

The present study investigated whether NAC reduces platelet activation by restoring HMA. The findings reconcile with this mechanism as recently described and suggest that NAC could be an effective antiplatelet agent in conditions where oxidative stress may increase the thrombotic risk.

## 2. Materials and Methods

### 2.1. Blood Collection and Plasma Preparation

The study was approved by the local institutional Ethics Committee and was performed according to the Declaration of Helsinki. Twenty-five healthy donors, both male and female (25–60 years of age) were enrolled, and all the participants provided written informed consent at the time of enrollment. None of the study participants had received any anti-inflammatory drug, nor drugs that could affect platelet function, in the previous 10 days. Venous blood from donors was collected in Vacutainer^®^ tubes containing 0.129 mol/L sodium citrate as an anticoagulant. Healthy donors had a platelet count between 250–350 × 10^3^/μL. Platelet-rich plasma (PRP) was obtained by centrifugation at 100× *g* for 10 min (no brake), and platelet-poor plasma (PPP) was obtained by centrifugation of PRP at 700× *g* for 15 min.

### 2.2. Plasma Protein Depletion

HSA and other high molecular weight plasma proteins (>30 kDa) were removed by centrifugation of plasma at 15,000× *g* for 30 min using centrifugal filters (Amicon^®^Ultra, Merck Life Science S.r.l., Milan, Italy).

### 2.3. Platelet Aggregation Assays

Platelet aggregation was measured with a PAP-8E Biodata^®^ optical aggregometer (Bio/Data Corporation, Sentinel Diagnostic, Milan, Italy). PRP (250 μL) was pre-incubated with NAC (25–100 μg/mL; Sigma-Aldrich S.r.l., Milan, Italy) or with the vehicle for different times (10, 20, 30 min) at 37 °C with constant stirring (1200 rpm). Aggregation was induced by the addition of different agonists, and monitored for 6 min as change in light transmittance. In another set of experiments, PRP was centrifuged at 700× *g*, and the platelet pellet was resuspended in 250 μL of plasma depleted of high molecular weight protein (>30 kDa) in the absence or presence of vehicle-treated HSA (Albutein^®^, Grifols Italia, S.p.A., Milan, Italy) or NAC-treated HSA. Aggregation was induced by the addition of collagen (8 μg/mL; Mascia Brunelli, Milan, Italy). Results are expressed as the area under the curve (AUC). 

### 2.4. Measurement of Thromboxane B2 and 12-Hydroxyeicosatetraenoic Acid

Six minutes after the addition of collagen to PRP, platelet aggregates were pelleted and plasma was collected. The levels of thromboxane B_2_ (TxB_2_) and 12-hydroxyeicosatetraenoic (12-HETE), the major arachidonic acid metabolites derived from cyclooxygenase and lipoxygenase pathways, respectively, and most abundant eicosanoids released during aggregation, were measured using a liquid chromatography-tandem mass spectrometry (LC-MS/MS) method previously developed and validated [31]. Briefly, after the addition of deuterated d4-TxB_2_ and d8-12-HETE as internal standards and the solid-phase extraction of serum samples, analytes were resolved using reversed-phase C18 column and quantified using negative ion electrospray ionization-tandem mass spectrometry. 

### 2.5. Measurement of Glutathione

GSH levels were measured in plasma and platelets after 30 min incubation of PRP with and without NAC, exogenous GSH, or GSH ethyl ester (GSHO-Et). Platelets were then isolated by centrifugation (700× *g* for 15 min) and washed with phosphate buffered saline (PBS). Intracellular and plasma GSH levels were quantified using a LC-MS/MS method previously developed and validated by us [32]. Briefly, chromatographic separation was conducted on a Luna PFP analytical column (100 × 2.0 mm, 3 µm, Phenomenex, Castel Maggiore, Bologna, Italy), eluted at 35 °C under isocratic conditions at 200 µL/min by 1% methanol in ammonium formate 0.75 mmol/L adjusted to pH 3.5 with formic acid. Analysis was performed by an Accela chromatographic system coupled with a triple quadrupole mass spectrometer TSQ Quantum Access (Thermo Fisher Scientific, Rodano, Milan, Italy) using an electrospray ionization source in positive ion mode. The transitions used in the multiple reaction monitoring were *m*/*z* 308.1→*m*/*z* 76.2 + 84.2 + 161.9. Data were obtained by comparison with calibration curves using GSH standard solutions (Sigma-Aldrich S.r.l.). The intra- and inter-day CVs % obtained with standard samples were <5%. The limits of detection were 0.031 µmol/L. Levels of GSH were expressed as μmol/L concentration and data are reported as mean ± SD.

### 2.6. Measurement of Cysteinylated form of Albumin

HSA-Cys was measured by mass spectrometry analysis of intact proteins in plasma, as previously described [33]. Briefly, platelets were removed and plasma proteins were separated by liquid chromatography on a reversed-phase Phenomenex LC column Jupiter—C4 (150 × 2 mm, i.d. 5 µm, 300 Å, Milan, Italy) and analyzed by a triple-quadrupole mass spectrometer (Finnigan TSQ Quantum Ultra, ThermoQuest, Milan, Italy) equipped with an Electrospray Finnigan Ion Max source. MagTran software (Mag-Tran 1.03b2) was used for spectra deconvolution and calculation of the relative abundance of HSA-Cys.

### 2.7. Albumin Cysteinylation

HSA-Cys was obtained following incubation of HSA (Albutein^®^) with 17 mmol/L cystine at 37 °C for 24 h. The solution was then filtered through Millex-HV (0.45 μm; Merck Life Science S.r.l., Milan, Italy) [34]. 

### 2.8. Platelet Adhesion Assays

The 96-well microtiter plates were coated (overnight at 4 °C) by adding 100 μL per well either with collagen type I (100 μg/mL; Roche Diagnostic S.p.a. Monza, Monza Brianza, Italy) or human fibrinogen (100 μg/mL; Merck Life Science S.r.l.). The non-specific platelet adhesion was prevented by the pre-treatment of the wells with 200 μL 1% bovine serum albumin for 1 h at 37° C. Following three washing steps, 50 μL PRP (treated with 100 μg/mL NAC or vehicle for 30 min at 37 °C) were added to each well and incubated at 37 °C without stirring to allow platelet adhesion. After 1 h, non-adherent platelets were removed, the plate was washed, and adherent cells were quantified through the measurement of the acid phosphatase activity. Briefly, 150 μL of acid phosphatase substrate (100 mmol/L citrate buffer pH 5.4, containing 5 mmol/L p-nitrophenyl phosphate and 0.1% Triton X-100) was added to each well. After 1 h incubation at 37 °C, the reaction was stopped by the addition of 100 μL NaOH 2 N. The p-nitrophenol produced was then measured by reading the absorbance at 405 nm (Infinite M Plex, Tecan, Tecan S.r.l., Cernusco sul Naviglio, Milan, Italy). A set of experiments was performed using tirofiban (Sigma-Aldrich S.r.l.), an αIIbβ3 antagonist. PRP was treated with tirofiban 5 μg/mL, for 30 min at 37 °C before the adhesion assay.

### 2.9. Measurements of Intracellular Calcium Mobilization

PRP was incubated with 2 μmol/L Fura-2 AM (abcam, Prodotti Gianni, Milan, Italy) for 1 h at 30 °C in the dark. Platelets were precipitated and subsequently resuspended in autologous PPP preincubated with vehicle or 100 μg/mL NAC for 30 min at 37 °C. A total of 100 μL per well of platelet suspension was added in a black wall, clear-bottom 96-well plate. Platelets were then stimulated by adding 0.5 μg/mL collagen, and the fluorescence was detected at 510 nm emission with excitation at 340 nm and 380 nm (Infinite M Plex, Tecan, Tecan S.r.l.). The fluorescence signal was monitored every 30 s for 6 min.

### 2.10. Determination of Reactive Oxygen Species Generation

The endogenous generation of reactive oxygen species (ROS) was measured by the intracellular oxidation of 2′,7′-dichlorofluorescin (DCFH; Sigma-Aldrich S.r.l.). A total of 100 μL PRP was incubated with non-fluorescent DCFH for 1 h at 37 °C with stirring. Platelets were precipitated and resuspended in autologous PPP previously treated with vehicle or 100 μg/mL NAC for 30 min at 37 °C, and stimulated with collagen (0.5 μg/mL). Intracellular DCFH was oxidized to fluorescent 2′,7′-dichlorofluorescein (DCF), and fluorescence was recorded for 6 min using Infinite M Plex Tecan (Tecan S.r.l.) by exciting the sample at 485 nm and measuring the resulting fluorescence at 535 nm.

### 2.11. Statistical Analysis

Data are expressed as mean ± SD. Differences between the groups were assessed by the Student *t*-test for single comparison or by the analysis of variance for repeated measures (ANOVA) and Dunnett’s or Tukey’s post hoc test, where indicated. A *p*-value < 0.05 was considered significant.

## 3. Results

### 3.1. Effect of N-Acetylcysteine on Platelet Aggregation and Arachidonic Acid Metabolite Generation

**Figure 1:**
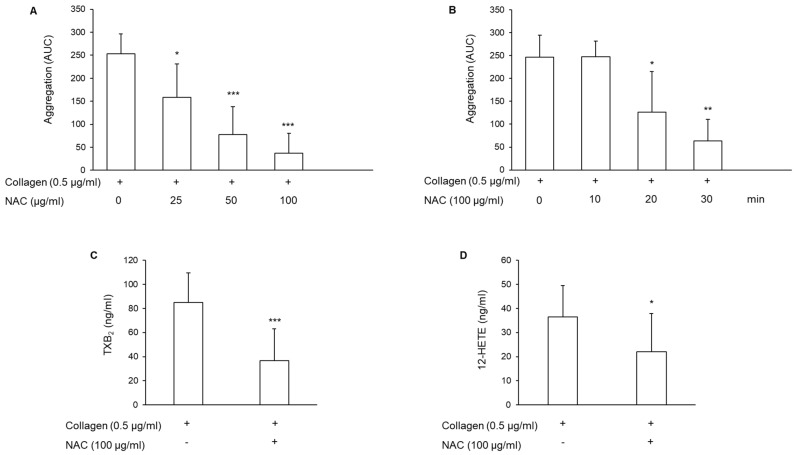
Effect of NAC on platelet aggregation and synthesis of arachidonic acid metabolites. PRP was incubated with NAC for 30 min at 37 °C, and subsequently, platelets were stimulated with collagen. (**A**) Concentration-dependent inhibition of aggregation induced by 0.5 μg/mL collagen after pre-treatment with NAC, *n* = 5. (**B**) Time-dependent inhibition of aggregation induced by collagen after pre-treatment with 100 μg/mL NAC, *n* = 4. (**C**) Levels of TxB_2_ and (**D**) 12-HETE measured in PRP pre-treated with NAC and stimulated with collagen. *n* = 9; * *p* < 0.05, ** *p* < 0.01, *** *p* < 0.001 vs. collagen-stimulated PRP for ANOVA and Dunnett’s post hoc test (**A**,**B**) and for Student *t*-test (**C**,**D**).

PRP obtained from healthy subjects was pretreated with different concentrations of NAC for different times, as indicated in Figure 1. Subsequently, platelets were stimulated with 0.5 μg/mL collagen, and aggregation was monitored for 6 min. NAC inhibited collagen-induced platelet aggregation in a concentration- and time-dependent manner (Figure 1A,B). An almost complete inhibition was observed using 100 μg/mL NAC for 30 min (−85.77% ± 17.6%), so all subsequent experiments were performed using these conditions. The inhibition of platelet aggregation induced by NAC is not stimulus-dependent, as it was also observed with other stimuli like adenosine diphosphate (ADP) (244.50 ± 84.73 and 59.77 ± 63.88 using ADP 2 μmol/L in the absence or in the presence of NAC, respectively, *n* = 4; *p* < 0.02), and the thromboxane A_2_ (TxA_2_) analog U46.619 2 μmol/L (260.0 ± 21.24 and 56.50 ± 64.80 in the absence or in the presence of NAC, respectively, *n* = 4; *p* < 0.05). 

As platelet activation triggers the induction of the arachidonic acid cascade with the generation of TxA_2_, the main metabolite derived from the cyclooxygenase pathway, and of 12-HETE from the lipoxygenase pathway, these eicosanoids were measured as described in the Methods section. As TxA_2_ is unstable, the stable metabolite thromboxane B_2_ (TxB_2_) was measured. The results show that NAC significantly reduced both TxB_2_ and 12-HETE synthesis induced by collagen (Figure 1C,D).

### 3.2. Involvement of the Intracellular Glutathione in the Antiplatelet Effect of N-Acetylcysteine 

**Figure 2:**
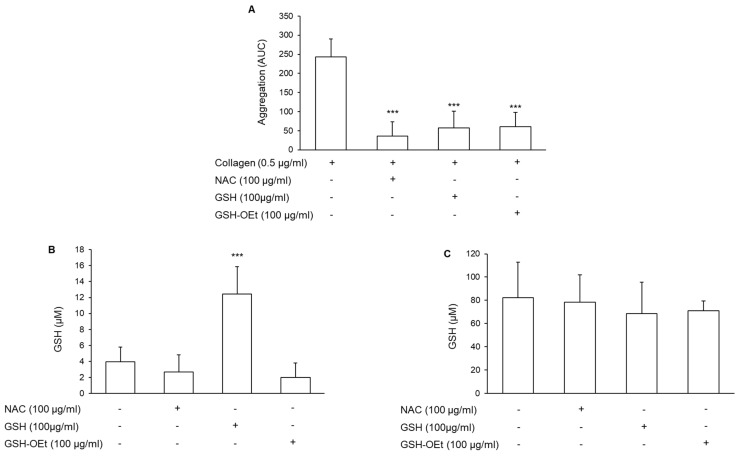
Effect of GSH, NAC, and GSH-OEt on platelet aggregation and on plasma and platelet GSH levels. PRP was incubated with NAC, GSH, or GSH-OEt for 30 min at 37 °C. (**A**) Platelet aggregation was induced by the addition of collagen and monitored for 6 min, *n* = 5. *** *p* < 0.001 vs. collagen-stimulated PRP. (**B**) Plasma and (**C**) intracellular GSH levels were measured by LC-MS/MS. *n* = 5 and *n* = 6 for plasma and platelet levels, respectively. *** *p* < 0.001 vs. basal condition for ANOVA and Dunnett’s post hoc test.

Most of the effects induced by NAC are attributed to its ability to generate GSH [35]. To verify this possibility, platelet aggregation was induced in collagen-treated PRP incubated with GSH (100 μg/mL) or with the membrane-permeable GSH-derivative GSH-OEt. Both compounds reduced platelet aggregation to an extent fully comparable to NAC (Figure 2A). As expected, a marked increase in GSH levels was detected in plasma after incubation with GSH exogenously added (Figure 2B). However, the incubation of PRP for 30 min with both NAC and GSH or GSHO-Et did not induce intracellular GSH accumulation (Figure 2C).

### 3.3. Effect of N-Acetylcysteine on the Restoration of HMA

**Figure 3:**
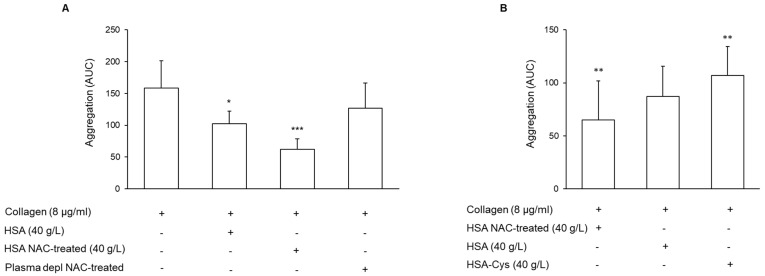
Effect of the different redox status of HSA on platelet aggregation. (**A**) Plasma depleted of high molecular weight proteins including HSA was incubated with or without HSA pre-treated with vehicle or NAC for 30 min at 37 °C. Platelet aggregation was induced by the addition of collagen and monitored for 6 min, *n* = 6. * *p* < 0.05, *** *p* < 0.001 vs. collagen-stimulated platelet. (**B**) HSA pre-treated with vehicle or NAC for 30 min at 37 °C or HSA-Cys was added to plasma depleted. Platelet aggregation was induced by the addition of collagen and monitored for 6 min, *n* = 5. ** *p* < 0.01 vs. platelets resuspended with plasma depleted containing HSA, for ANOVA and Dunnett’s post hoc test.

To address the hypothesis that NAC might act on platelet aggregation by restoring HMA, a series of experiments was performed using plasma depleted of high molecular weight proteins including HSA. HSA (Albutein^®^) exogenously added, at a physiological concentration (40 g/L), to the depleted plasma, reduced platelet aggregation induced by collagen (Figure 3A). This commercially available solution of HSA contained about 37% ± 1.86% of HSA-Cys, and the treatment with NAC (100 μg/mL for 30 min) reduced the HSA-Cys level to 14.03% ± 1.24% [36]. The addition of NAC-pre-treated HSA to plasma depleted further reduced the platelet aggregation induced by collagen (Figure 3A). In particular, NAC-pre-treated HSA significantly reduced platelet aggregation compared to that measured with vehicle-treated HSA (*n* = 6; *p* = 0.01 paired *t*-test). To better clarify the role of HMA on platelet aggregation, plasma depleted was treated with NAC in the absence of HSA. The results showed a modest, not statistically significant, inhibition of platelet aggregation (Figure 3A). Conversely, the addition of the in vitro obtained Cys-HSA to the plasma depleted induced an increase in platelet aggregation with respect to that detected after the addition of HSA (Figure 3B). Overall, these results support the anti-platelet role of HMA.

**Figure 4:**
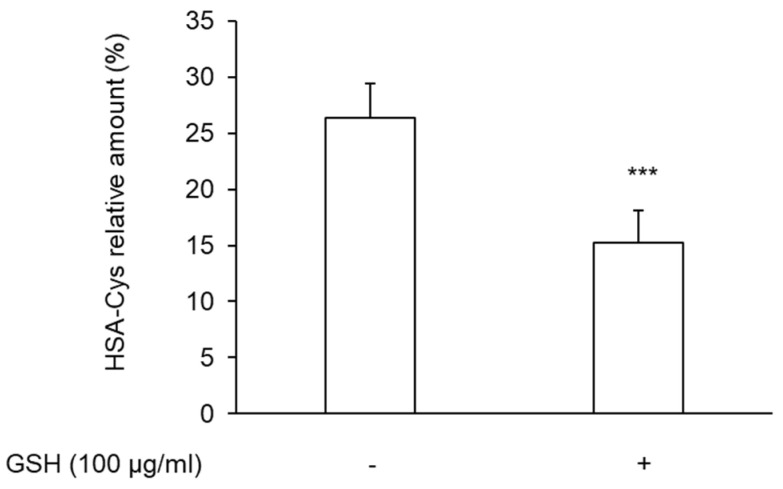
Levels of HSA-Cys in PRP. PRP was incubated with 100 μg/mL GSH for 30 min at 37 °C. Platelets were removed, and the levels of HSA-Cys were measured in plasma mass analysis. *n* = 6. *** *p* < 0.001 for Student *t*-test.

In addition, results suggest that the anti-platelet effect of exogenously added GSH is likely attributable to the reduction in the levels of HSA-Cys. Indeed, the analysis of plasma treated with GSH showed a progressive decrease in HSA-Cys plasma levels after incubation with 100 μg/mL GSH, reaching a reduction in 37.29% ± 8.32% at 30 min (Figure 4).

### 3.4. Effect of Cysteine on Platelet Aggregation 

**Figure 5:**
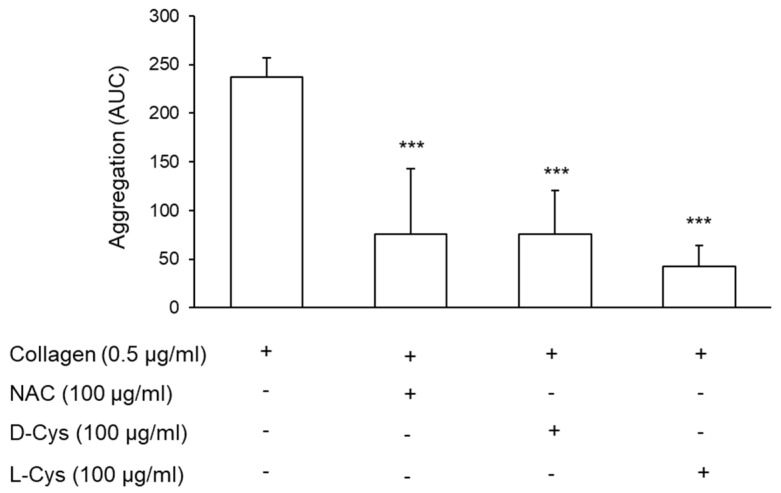
Effect of D- and L-Cys on platelet aggregation. PRP was incubated with NAC, D-Cys, or L-Cys for 30 min at 37 °C. Platelet aggregation was induced by the addition of collagen and monitored for 6 min, *n* = 4. *** *p* < 0.001 vs. collagen-stimulated PRP, for ANOVA and Dunnett’s post hoc test.

As previously reported, cysteine restores HMA by reducing the HSA-Cys form [33]. The pre-treatment of PRP with both D- and L-cysteine (Cys) in the same conditions performed with NAC reduced platelet aggregation promoted by collagen to an extent similar to that observed with NAC (−66.6% ± 29.9%, −67.0% ± 21.4%, −81.85% ± 10.0%, for NAC, D-Cys, and L-Cys, respectively) (Figure 5).

### 3.5. Effect of NAC-Pretreated PRP on Platelet Adhesion 

**Figure 6:**
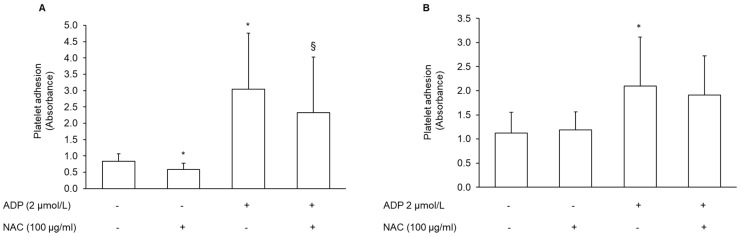
Effect of NAC on platelet adhesion to extracellular matrices. PRP was incubated with vehicle or NAC for 30 min at 37 °C, and added to (**A**) collagen- or (**B**) fibrinogen-coated plates. Platelets were incubated for 1 h at 37 °C. Non-adherent platelets were removed, and the adherent platelets were quantified through the measurement of acid phosphatase activity. Data are expressed as absorbance, *n* = 7. * *p* < 0.05 vs. basal, § *p* < 0.05 vs. ADP-stimulated platelets for the Student *t*-test in the comparison of the NAC-treated sample vs. the respective sample without NAC.

Platelet adhesion was evaluated both on collagen and fibrinogen surfaces. The pre-treatment of PRP with NAC significantly inhibited the adhesion to collagen. Conversely, platelet adhesion to fibrinogen was not affected (Figure 6A,B). Adhesion was also measured after the addition of 2 μmol/L ADP. ADP increased platelet adhesion to both the collagen and fibrinogen surface (300.39% ± 265.23%; *n* = 7, and 81.09% ± 34.45% *n* = 7, for collagen and fibrinogen matrix, respectively). Similar to what was found in basal conditions, also after stimulation with ADP, NAC reduced platelet adhesion to collagen, but not to the fibrinogen matrix (Figure 6A,B). The pre-treatment of platelets with tirofiban, a selective αIIbβ3 receptor inhibitor, nearly completely inhibited platelet adhesion to fibrinogen coated wells (−82% ± 7.87%, *n* = 5).

### 3.6. Effect of NAC-Pretreated Plasma on Intracellular Calcium Mobilization 

**Figure 7:**
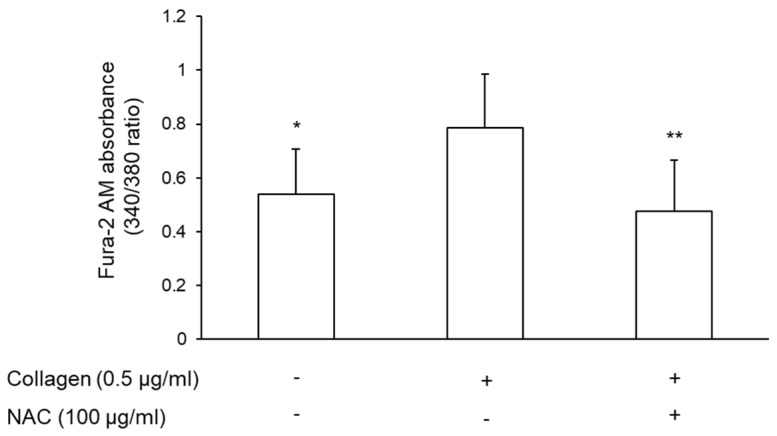
Effect of NAC on intracellular calcium mobilization. PRP was incubated with 2 μM Fura-2 AM. Platelets were precipitated and resuspended in plasma pre-treated with vehicle or NAC for 30 min at 37 °C. Platelets were stimulated with collagen (0.5 μg/mL) for 6 min. Data are expressed as absorbance of Fura-2 AM, *n* = 4. * *p* < 0.05 and ** *p* < 0.01 vs. collagen-stimulated platelets for ANOVA and Dunnett’s post hoc test.

Calcium mobilization is critical for platelet activation and aggregation. Indeed, an increase in intracellular calcium ([Ca^2+^]i) triggers several signaling pathways, whilst its decrease reduces platelet activation [37]. Therefore, it was evaluated whether the resuspension of platelets with plasma pre-treated with vehicle or NAC affected [Ca^2+^]i mobilization. Stimulation of platelets with collagen induced a marked increase in [Ca^2+^]i levels (49.15% ± 17.34%). NAC-pretreated plasma significantly reduced collagen-induced [Ca^2+^]i mobilization (Figure 7).

### 3.7. Effect of NAC-Pretreated PRP on Reactive Oxygen Species Generation 

**Figure 8:**
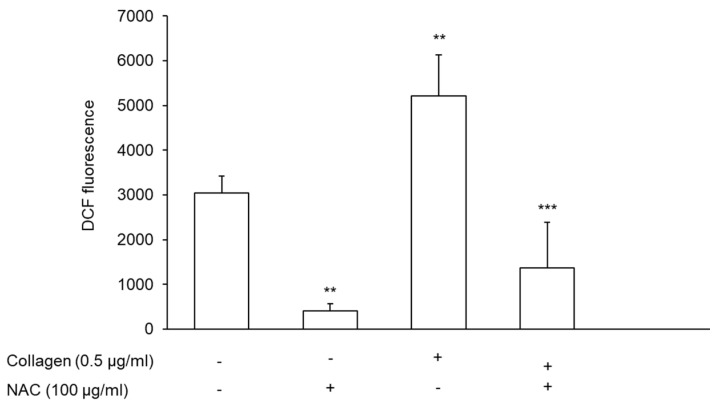
Effect of NAC on the endogenous generation of ROS by platelets. PRP was incubated with DCFH for 1 h at 37 °C. Platelets were precipitated and resuspended in plasma pretreated with vehicle or NAC for 30 min at 37 °C and stimulated with 0.5 μg/mL. Fluorescence was detected 6 min after the addition of collagen. Data are expressed as absorbance of intracellular DCF *n* = 5. ** *p* < 0.01 vs. basal; *** *p* < 0.001 vs. collagen-stimulated platelets for ANOVA and Tuckey’s post hoc test.

Several experimental and clinical studies have shown that platelets are able to generate ROS [38]. The stimulation of platelets with collagen induced a marked increase in ROS generation (71.25% ± 20.42%; *n* = 5) and the preincubation of plasma with NAC significantly reduced ROS endogenously generated by platelets both in unstimulated and stimulated conditions (Figure 8).

## 4. Discussion

This paper showed that NAC is able to reduce platelet activation through a mechanism recently described, which is based on its disulfide-breaking activity and involves the reduction in HSA-Cys. In particular, NAC-mediated HMA-regeneration affects platelet function in terms of aggregation, synthesis of arachidonic acid metabolites, adhesion, the production of oxygen radicals, and calcium mobilization.

A number of papers have shown that plasma HSA can influence platelet activity [21,23,39,40,41]. More recently, it has been shown that HSA is subjected to different oxidative modifications that can affect its properties. In particular, oxidative modifications of Cys34 have been reported in vivo, and increased levels of these oxidized forms have been detected during aging and in pathological conditions associated with oxidative stress [18]. Indeed, the oxidative forms of HSA induce oxidative stress, promote inflammation, and trigger platelet activation [25].

A previous study has shown that in vitro treatment of HSA with NAC reduced the levels of its cysteinylated form to regenerate the native form [33]. This study showed that the treatment of PRP with NAC inhibited collagen-induced platelet aggregation in a concentration- and time-dependent manner. This inhibition was not agonist-specific, as ADP- and U46.619-induced aggregation was similarly affected. It has been previously shown by Gibson et al. that NAC inhibited ADP- and thrombin-induced platelet aggregation in association with an increase in intracellular GSH accumulation [42]. In our experimental conditions, no increase in GSH levels was detected in platelets exposed to NAC, likely due to the short incubation time. However, inhibition of platelet aggregation was also detected following treatment of PRP with GSH. Indeed, similar to NAC and Cys, we showed that GSH also reduced the HSA-Cys levels.

Similarly, cysteine is another compound able to restore HMA by reducing the HSA-Cys content [33]. Accordingly, the treatment of PRP with D- and L-Cys also inhibited platelet aggregation, similarly to that found with NAC. 

The capacity of HMA to inhibit platelet aggregation has been attributed to several mechanisms that include a reduction in the conversion of arachidonic acid to the pro-aggregating agent TxB_2_, and an increase in the formation of PGD_2_, which inhibits platelet aggregation [39]. Accordingly, a significant inhibition of the release of the arachidonic acid metabolite TxB_2_ was detected, suggesting a potential role of this metabolite in the effect observed after exposure of PRP to NAC. In addition, a marked reduction in 12-HETE was also shown. The role of 12-HETE in platelet function is debated, and both pro-thrombotic and anti-thrombotic effects have been reported [43]. Nevertheless, the presence of 12-HETE increases thrombin-induced platelet aggregation [44]. The mechanism(s) by which NAC-pretreated plasma reduces the synthesis of these metabolites of arachidonic acid is unknown; even if our data suggest that the inhibitory effect of NAC-regenerated HMA on platelet aggregation is upstream of the cyclooxygenase and lipoxygenase pathways. In line with this assumption, it has been noted that NAC reduces the mobilization of arachidonic acid in macrophages [45]. 

The antioxidant activity of HMA is mediated by the thiol group, and although oxidized forms of albumin have been shown to induce platelet activation and promote platelet-endothelium interaction [24,25], no information is available about the ability of regenerated HMA, obtained after NAC treatment, to modulate platelet function. In order to define the possible mechanism responsible for the inhibitory effect shown by NAC, platelets were precipitated from PRP and resuspended in autologous plasma depleted of high molecular weight proteins including HSA, pretreated with or without HSA and with or without NAC. The results showed a marked reduction in collagen-induced platelet aggregation after the addition of HSA and NAC to the depleted plasma. In contrast, no inhibition of aggregation was detected after resuspension of platelets with NAC-treated-depleted plasma in the absence of HSA. Finally, platelets resuspended in depleted plasma containing HSA-Cys showed an increased aggregation with respect to that of HSA or HSA NAC-treated. These results support the role of the reduced form of HSA in the inhibition of platelet aggregation detected after NAC-treatment.

It is also possible to speculate that the reduced form of HSA could block the nucleotide surface receptor P2Y12 receptor by acting at the level of free cysteines present on it. Nucleotide surface receptors expressed on platelets play a crucial role in platelet function and among them, the activation of the P2Y12 receptor shows a central role in platelet aggregation [46]. Recently, it has been shown that this receptor has two free cysteines in its extracellular domain, which are the target of the thiol reagent, and it has been suggested that antiplatelet drugs such as clopidogrel inactivate the receptor through the formation of disulfide bridges [47].

In the hemostatic system, the first event that occurs after a vascular injury is the adhesion of platelets to the subendothelium, an essential process to counteract blood loss. However, an excessive or inappropriate adhesion can lead to the uncontrolled formation of a thrombus with consequent vascular occlusion and ischemia [48]. The first step in the adhesion process is the interaction of the integrins and non-integrin proteins such as glycoproteins (GPs) expressed on the platelet membrane with the subendothelial collagen, the major constituent of the extracellular matrix. The adhesion to collagen involves several receptors such as the integrins α2bβ1 or GPIa/IIa, and αIIbβ3, also known as GPIIb/IIIa, and GPIb and GPIV [49]. Integrin αIIbβ3 plays a pivotal role in the formation of the fibrin cloth because it binds proteins containing the RGD sequence including fibrinogen, its major ligand [50]. The incubation of PRP with NAC significantly inhibited platelet adhesion to the collagen surface without affecting their adhesion to fibrinogen. In contrast, the pretreatment of platelet with tirofiban, which selectively inhibits the αIIbβ3 receptor, markedly reduced platelet adhesion to fibrinogen. These findings suggest that NAC-treated HSA differentially modulates integrin activation without affecting the conformational change required to bind the fibrinogen with high affinity. 

Platelet activation is triggered by several agonists, which despite acting on different platelet receptors and activating different signaling pathways, all lead to an increase in [Ca^2+^]i [51]. In addition, it is known the crosstalk between calcium and ROS, indeed an increase in calcium levels, can increase the ROS production and an increase in ROS generation can trigger an increase in [Ca^2+^]i [52]. This study showed that the pretreatment of plasma with NAC fully prevents the increase in [Ca^2+^]i induced by collagen. 

It has been previously suggested that the inhibition of ROS generation is not a direct effect of NAC since it is a weak antioxidant in its own right [42]. In addition, this study rules out a role mediated by an intracellular increase of GSH, and suggests that the antioxidant action of NAC is dependent on its ability to regenerate the native form of HSA, the main antioxidant in plasma.

## 5. Conclusions

This paper showed that NAC reduces platelet activation through a mechanism recently described that involves the restoration of the native form of HSA. Although it is commonly accepted that NAC acts as a precursor for GSH biosynthesis or as a scavenger of reactive oxygen species, these mechanisms cannot explain all the activities of NAC. Indeed, it has been evidenced that while NAC is effective in restoring GSH under conditions of deficiency, it is ineffective in increasing GSH in normal conditions [53]. Moreover, NAC per se is a weak antioxidant [42], but can act indirectly through the formation of other compounds. Indeed, NAC is able to break the disulfide bonds of several proteins, and among them, it can reduce the mixed disulfide forms of HSA, restoring its native antioxidant form. Through this mechanism, NAC is able to modulate multiple platelet function, reducing aggregation, adhesion, intracellular calcium mobilization, and ROS generation. 

Overall, these results open a new perspective on the therapeutic use of NAC in conditions of oxidative stress associated with platelet activation.

## Data Availability

Data collected in the study will be made available using the data repository Zenodo (https://zenodo.org (accessed on 2 February 2022)) with restricted access upon request to direzione.scientifica@ccfm.it.
